# MPore: database-driven identification of active methyltransferases in prokaryotic genomes from nanopore sequencing

**DOI:** 10.1093/bioadv/vbag077

**Published:** 2026-03-24

**Authors:** Azlan Nisar, Lars Vogelgsang, Sebastian Fuchs, Klaus Pfeffer, Birgit Henrich, Alexander Dilthey

**Affiliations:** Institute of Medical Microbiology and Hospital Hygiene, University Hospital Düsseldorf, Heinrich Heine University Düsseldorf, Universitäts str. 1, Düsseldorf, North-rhine-westphalia, 40225, Germany; Center for Digital Medicine, Heinrich Heine University Düsseldorf, Henkelstr. 230, Düsseldorf, North-rhine-westphalia, 40599, Germany; Institute of Medical Microbiology and Hospital Hygiene, University Hospital Düsseldorf, Heinrich Heine University Düsseldorf, Universitäts str. 1, Düsseldorf, North-rhine-westphalia, 40225, Germany; Institute of Medical Microbiology and Hospital Hygiene, University Hospital Düsseldorf, Heinrich Heine University Düsseldorf, Universitäts str. 1, Düsseldorf, North-rhine-westphalia, 40225, Germany; Institute of Medical Microbiology and Hospital Hygiene, University Hospital Düsseldorf, Heinrich Heine University Düsseldorf, Universitäts str. 1, Düsseldorf, North-rhine-westphalia, 40225, Germany; Institute of Medical Microbiology and Hospital Hygiene, University Hospital Düsseldorf, Heinrich Heine University Düsseldorf, Universitäts str. 1, Düsseldorf, North-rhine-westphalia, 40225, Germany; Institute of Medical Microbiology and Hospital Hygiene, University Hospital Düsseldorf, Heinrich Heine University Düsseldorf, Universitäts str. 1, Düsseldorf, North-rhine-westphalia, 40225, Germany; Center for Digital Medicine, Heinrich Heine University Düsseldorf, Henkelstr. 230, Düsseldorf, North-rhine-westphalia, 40599, Germany

## Abstract

**Motivation:**

In prokaryotic genomes, methylation is an important epigenetic modification that regulates the uptake of foreign DNA; it can also contribute to replication or virulence. We present MPore, a novel method for the database-driven detection of active methyltransferases and their associated target site recognition motifs from Nanopore R10 sequencing data of prokaryotic isolates. In contrast to existing methods, which typically start with the *de novo* identification of differentially methylated sequence motifs, MPore starts by identifying potential methyltransferase genes by homology search against REBASE; activity is then assessed through a regularized logistic regression model of observed genome-wide methylation patterns, integrating motif and genomic sequence context information.

**Results:**

On two benchmarking datasets, 10 bacterial monocultures and two *Helicobacter pylori* genomes with complex methylation patterns, MPore achieved a combined recall of 93% and a combined PPV of 96%, outperforming Nanomotif (81%/91%), Modkit (66%/4%), and Snappy (89%/50%). Further validation on a well-characterized dataset of *Mycoplasma hominis* isolates showed perfect agreement with wet-lab-based validation results and demonstrated that MPore could complement REBASE information by disambiguating the specific methylated base in a motif with multiple potential methylation sites. MPore automatically produces integrated visualizations of the identified methyltransferases and observed methylation patterns; the tool is implemented as a user-friendly Snakemake pipeline.

**Availability and implementation:**

MPore is freely available under the MIT license at https://github.com/DiltheyLab/MPore.

## 1 Introduction

DNA methylation is an epigenetic process that involves the addition of methyl groups to the DNA bases cytosine or adenine (Feng and Lou 2019). In prokaryotes, the three main types of methylation are 4mC, 5mC, and 6mA; while prokaryotic methylation was traditionally understood to play an important role mainly in the context of defense against invading elements and RM (restriction modification) systems, it is now well-established that it also plays an important regulatory role (Seong *et al.* 2021), contributing to important phenotypes such as cell motility, virulence, and replication (Kumar *et al.* 2012, Putnam 2016, Kumar *et al.* 2018, Oliveira *et al.* 2020). The single-molecule long-read sequencing technologies Oxford Nanopore and Pacific Biosciences are natively methylation-aware and enable the detection of the methyltransferases and their target site recognition motifs in large numbers of individual prokaryotic genomes (Blow *et al.* 2016, Tisza *et al.* 2023). In general, there are two complementary approaches for identifying active methyltransferases (MTases). The “*de novo*” approach starts by identifying the sequence motifs that exhibit evidence for differential methylation, followed by searching the interrogated genome for candidate methyltransferases that may account for the observed patterns; the *de novo* approach has been implemented in tools like Snappy (Konanov *et al.* 2025) or Nanomotif (Heidelbach *et al.* 2024). The “database-driven” approach starts by identifying putative methyltransferase, e.g. by homology search against databases like REBASE, followed by an activity assessment based on interrogating the target sites of the identified candidates for evidence of differential methylation. One disadvantage of the database-driven approach is that it is limited by the methyltransferases and the target motifs present in the utilized reference database; on the other hand, it can integrate prior information on complex methyltransferase target motifs, which may be difficult to detect *de novo*, and account for overlaps and nesting between the target motifs of different methyltransferases. To our knowledge, there are no integrated user-friendly implementations of the database-driven approach. Here we present MPore, a user-friendly method for the database-driven identification of active methyltransferases in prokaryotic genomes from Oxford Nanopore R10 data. MPore takes as input genome assemblies and Nanopore POD5 signal data for a set of isolates; it identifies candidate methyltransferases by homology search against REBASE; genome-wide methylation is inferred from the Nanopore signal data; and a statistical model is used to assess activity of the candidate methyltransferases, based on the methylation status of sequenced isolates at the target sites of the candidate enzymes. The output generated by MPore includes visualizations that show the identified candidate enzymes, their recognition motifs, and the methylation status at the relevant genomic positions.

## 2 Methods

### 2.1 Implementation

**Figure 1:**
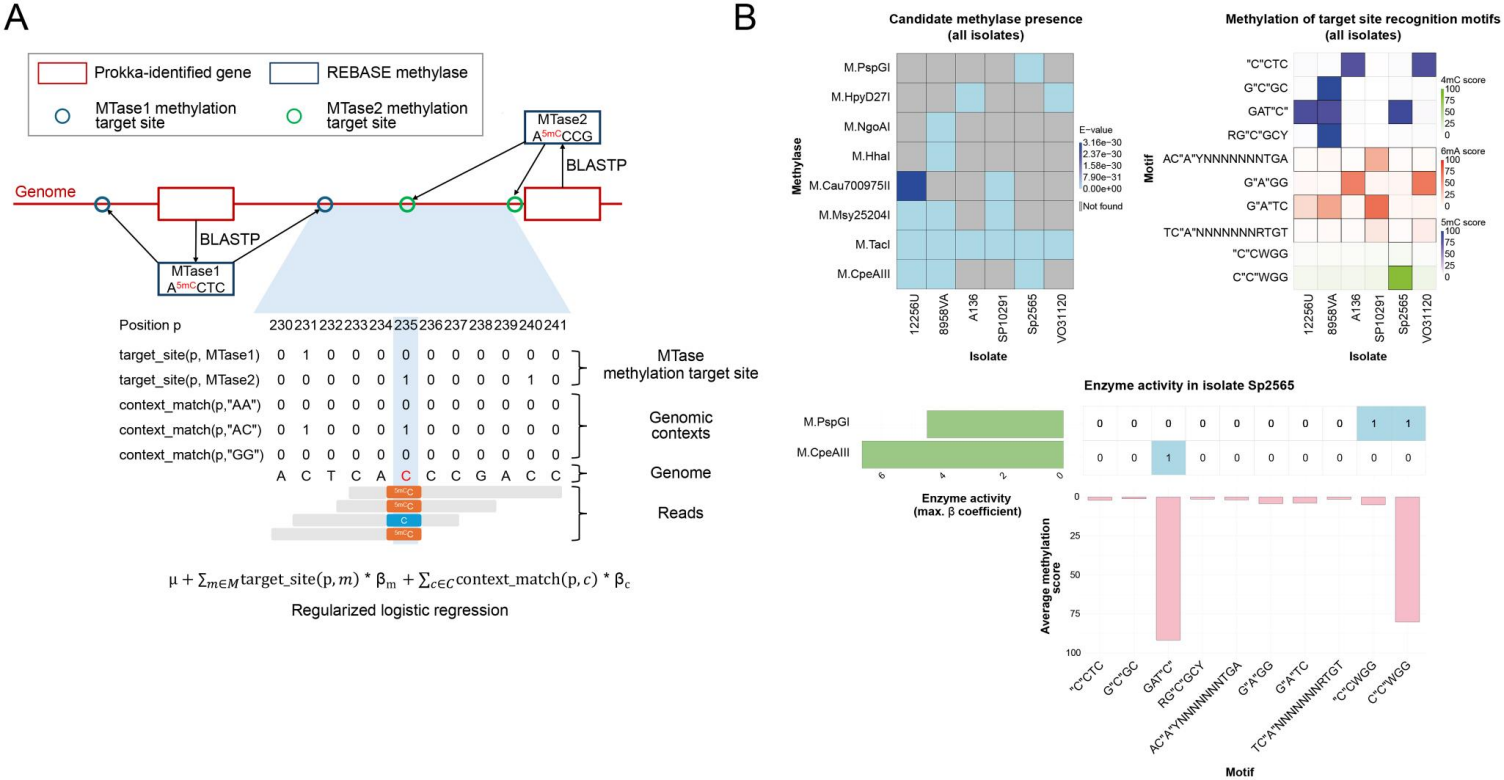
MPore algorithm and default plots. (A) Overview of the MPore algorithm. Candidate methyltransferases are identified by carrying out a REBASE search of Prokka-identified genes against the REBASE gold-standard dataset of methyltransferases. Methyltransferase target sites (visualized by the “target_site”-labeled rows in the matrix of the bottom part of the panel) are identified by mapping the candidates’ target site recognition motifs against the genome. Genome-wide methylation calling from Oxford Nanopore read data is performed using Dorado and modkit (visualized by the background color of the “C” bases in the shown aligned sequencing reads), and methyltransferase activity is assessed using a regularized logistic regression approach (glmnet), modeling observed methylation of sequencing reads at individual genomic positions conditional on the activity of the methyltransferase candidates targeting the position ($\beta_{m}$) as well as genomic sequence context effects ($\beta_{c}$). (B) Illustration of default plots automatically generated by MPore, at the example of a cohort of 6 *Mycoplasma hominis* isolates (see text for details). The top-left plot shows methyltransferase candidates identified for the analyzed isolates and the respective E-values of the BLAST search against REBASE. The top-right plot shows, for each methylation target site recognition motif and each isolate, the empirically achieved average methylation score. The bottom plot shows methyltransferase activity (i.e. after application of the regularized logistic regression model) for isolate SP2565; the binary matrix at the center of the plot shows the association between target site recognition motifs and methyltransferase candidates, the bar plot below the matrix shows the empirically achieved average methylation score at the corresponding motifs’ target sites, and the bar plot to the left of the matrix shows the results of the enzyme activity analysis (i.e. for each methyltransferase candidate, the plot shows the maximum achieved $\beta_{m}$ coefficient over all target site recognition motifs associated with the candidate after the motif disambiguation step).

MPore is implemented as a user-friendly Snakemake pipeline (Mölder *et al.* 2021) and available under the MIT license. Mandatory input arguments include paths to the genome assembly and Nanopore POD5 signal data for each isolate to be characterized. An MPore analysis (Fig. 1) comprises the following steps: (i) Identification of methyltransferase candidates: Coding sequences (CDS) in the input assemblies are identified using Prokka (Seemann 2014). For each identified CDS, a BLASTP (Camacho *et al.* 2009) homology search is carried out against the REBASE (Roberts *et al.* 2023) gold-standard set of methyltransferases, which only includes enzymes for which the recognition motifs and modification sites have been validated by both computational and experimental methods (Roberts *et al.* 2023). Hits with an *E*-value < $e^{-25}$ are considered as candidates and taken forward for further analysis. (ii) Methyltransferase target site identification: For each input assembly, target sites of the identified candidate methyltransferase are determined by mapping their target site recognition motifs, which may contain IUPAC ambiguity characters, against the assembly. The target sites of a methyltransferase are the genomic locations that are expected to be methylated if the methyltransferase is active. If the associated REBASE entry of a methyltransferase candidate does not specify which specific positions within the recognition motif are methylated (“methylation target sites”), the candidate is, prior to the identification of genomic target sites, replaced with *k* disambiguated candidate entries that each specify a single methylation target site within their motif (where *k* is the number of potential methylation target sites in the motif of the original REBASE entry; see Note S1, available as supplementary data at *Bioinformatics Advances* online for details). (iii) Methylation calling: The methylation status of each A and C site in the input genomes is determined using Dorado (https://github.com/nanoporetech/dorado) and modkit (https://github.com/nanoporetech/modkit), separately for the different types of methylation (4mC, 5mC, 6mA), and keeping track of the strand on which the base was recorded. (iv) Statistical assessment of methyltransferase activity: Observed methylation levels at individual genomic positions are modeled conditional on genomic sequence context and the activity of the identified candidate methyltransferases in a regularized logistic regression framework [glmnet (Friedman *et al.* 2010)]. Specifically, the probability that a sequenced nucleotide at a genomic position p is methylated is modeled using a linear predictor of the form


$$\mu +\sum_{m\;\in M}\text{target}\_\text{site}(p,m)\times \beta_{m}+\sum_{c\;\in C}\text{context}\_\text{match}(p,c)\times \beta_{c}$$

where $\beta_{m}\;$reflects the activity of methyltransferase m; $\beta_{c}$, the effect of genomic sequence context c; target_site(p,m) and context_match(p,c) are indicator functions specifying whether p is a target site of m and embedded in genomic context c. To avoid collinearity, methyltransferases with identical target site recognition motifs are merged. The regularization parameter λ for glmnet is set using 10-fold cross-validation. In single-isolate mode, each isolate is analyzed independently; in cross-isolate mode, multiple isolates are combined in the same analysis for a pooling of genomic sequence context effects, using an additional isolate-specific intercept $\mu_{i}$ in the linear predictor for the isolate i. See Note S1, available as supplementary data at *Bioinformatics Advances* online for a full description of the statistical model. (v) Visualization: As a last step, integrated visualizations of the identified methyltransferases, their target site recognition motifs, the methylation status at identified active sites, and the results of the statistical analysis are generated in a per-isolate manner and across the complete cohort of analyzed isolates.

### 2.2 Evaluation

To demonstrate MPore, we analyzed three datasets of Nanopore R10-sequenced bacterial isolates: 10 bacterial monocultures (Heidelbach *et al.* 2024), as well as two *Helicobacter pylori* strains (HPJ99 and HP26695) with complex methylomes (Krebes *et al.* 2014), with ground truth (Table S1, available as supplementary data at *Bioinformatics Advances* online) regarding active MTases and their recognition sequences. In addition, we also included six *Mycoplasma hominis* isolates with previously well-characterized methylation patterns (Vogelgsang *et al.* 2023). We benchmarked MPore against Nanomotif (Heidelbach *et al.* 2024), Modkit, and Snappy (Konanov *et al.* 2025), state-of-the-art methods for the *de novo* detection of methylation motifs. Exact command-line usage for motif analysis for each *de novo* approach is provided in Note S2, available as supplementary data at *Bioinformatics Advances* online (“Parameter settings for *de novo* approaches”). For MPore, a methyltransferase m was considered active if $\beta_{m}>2.021$; this threshold was determined based on a subset of 3 bacterial monoculture isolates (with MPore executed in cross-isolate mode), which were excluded from further benchmarking. See Note S2, available as supplementary data at *Bioinformatics Advances* online, for details and dataset accessions.

## 3 Results

The validation dataset for the 7 bacterial monocultures remaining for benchmarking comprised 23 methyltransferases with associated target site recognition motifs (Table S1, available as supplementary data at *Bioinformatics Advances* online; in the following, methyltransferases and target site recognition motifs in different isolates are always counted separately, as well as methyltransferases and target site recognition motifs associated with different methylation types). Executed in cross-isolate mode, MPore detected 23 genomic loci that mapped onto 30 REBASE methyltransferase genes with 24 target site recognition motifs; of these, 23 were classified as active (Table S2, available as supplementary data at *Bioinformatics Advances* online), translating into a recall of 100% and a PPV of 100% (Table 1). On these isolates, Nanomotif achieved the same performance as MPore; Snappy achieved a similar recall (97%) but lower PPV (38%); and Modkit exhibited lower recall (69%) and much lower PPV (2%).

**Table 1:** Benchmarking results.

	Bacterial monocultures		*H. pylori*	
	Recall	PPV	Recall	PPV
MPore (motif-based)	1.00	1.00	0.87	0.92
MPore (gene-based)	0.97	0.97	0.69	0.87
Nanomotif	1.00	1.00	0.64	0.81
Modkit	0.69	0.02	0.64	0.53
Snappy	0.97	0.38	0.82	0.78

Recall and PPV of MPore, Nanomotif, Modkit, and Snappy on two benchmarking datasets.

For the bacterial monocultures benchmarking dataset, cross-isolate and isolate-specific approaches yielded the same validation statistics for MPore (see Note S2, available as supplementary data at *Bioinformatics Advances* online). The validation dataset for two *H. pylori* strains with complex methylomes comprised 35 unique methyltransferases with 39 associated target site recognition motifs (Table S1, available as supplementary data at *Bioinformatics Advances* online). On these strains, MPore detected 54 genomic loci that mapped onto 31 REBASE methyltransferase genes with 37 target site recognition motifs (Table S2, available as supplementary data at *Bioinformatics Advances* online); of these, 36 were classified as active, translating into 87% recall and 92% PPV for the detection of methylated motifs and 69% recall and 87% for the detection of methyltransferase genes (Table 1). On the *H. pylori* dataset, MPore outperformed all other evaluated methods in terms of both recall and PPV (Table 1). See Tables S1 and S3, available as supplementary data at *Bioinformatics Advances* online, for detections of all evaluated tools on the bacterial monocultures and *H. pylori* datasets.

On six *M. hominis* isolates, MPore (executed in cross-isolate mode) detected 18 putative methyltransferase-encoding genomic loci, mapping onto 19 REBASE methyltransferase genes with 27 target site recognition motifs. Of these, 12 were classified as active (Table S2, available as supplementary data at *Bioinformatics Advances* online). MPore results were fully consistent with the results on 5mC and 6mA methylation presented in Vogelgsang *et al.* (2023) (see Table S4, available as supplementary data at *Bioinformatics Advances* online), except for two instances in isolate SP2565, which were confirmed by wet-lab validation data (Vogelgsang *et al.* 2023). First, the MPore finding of 4mC, but not 5mC, methylation in the second “C” of motif CCWGG was consistent with inactivity of the restriction enzyme MvaI, which is inhibited by 4mC methylation of the second “C” of its recognition motif CCWGG (Butkus *et al.* 1985). Furthermore, absence of 5mC methylation was consistent with a lack of restriction by enzyme SgeI, activity of which is dependent on 5mC methylation of the same position (i.e. C^5m^CCWGG). Second, MPore did not detect a 6mA methylation signal in the motif GATC, which was confirmed by the lack of restriction by the G^6m^AATC-recognizing enzyme DpnI. The results for isolate SP2565 illustrated the utility of the methylation site disambiguation feature of the MPore algorithm; the MPore analysis showed that methyltransferase M.PspGI exhibited 4mC methylation activity only for the second of the two Cs in its target site recognition motif CCWGG (see Fig. 1B, bottom panel), thus complementing the information contained in REBASE, which lacks positional specificity for this motif. For the *M. hominis* benchmarking dataset, cross-isolate and isolate-specific approaches differed in three instances. In single-isolate mode, enzyme M.TacI in isolate SP10291 was classified as active, whereas its estimated activity remained below the threshold of 2.021 in cross-isolate mode. Furthermore, the enzyme M.HpyD27I was classified as inactive with respect to 5mC methylation in single-isolate mode for isolates A136 and VO31120, whereas it was found to be active in cross-isolate mode (see Table S5, available as supplementary data at *Bioinformatics Advances* online). In all instances, manual inspection of methylation target site methylation plots indicated accuracy of cross-isolate results; specifically, while individual target sites of M.TacI in SP10291 exhibited high methylation levels, the majority of target sites exhibited low methylation; and for M.HpyD27I in isolates A136 and VO31120, the majority of target sites exhibited high methylation levels (see Note S2, available as supplementary data at *Bioinformatics Advances* online).

## 4 Conclusion

We have presented MPore, the first integrated method for the database-driven identification of active methyltransferases in prokaryotic genomes. While not supporting the detection of entirely novel enzymes, MPore benefits from the increasing representation of the space of prokaryotic methyltransferase genes in databases like REBASE. On two validation datasets, MPore achieved the highest combined recall (93%) and PPV (95%) when compared to three state-of-the-art methods for *de novo* identification of methylated motifs. In conclusion, MPore is a novel tool for the interrogation of the prokaryotic methylome with a database-driven approach.

## Supplementary Material

vbag077_Supplementary_Data

## Data Availability

All data utilized for the implementation and evaluation of MPore is available in Supplementary Note 2, including their corresponding download links, sites, and accession numbers.
