# Supplementary material for: MPore: database-driven identification of active methyltransferases in prokaryotic genomes from nanopore sequencing
Source: Bioinform Adv. 2026 Mar 24;6(1):vbag077. doi: 10.1093/bioadv/vbag077 (PMC13189711; doi:10.1093/bioadv/vbag077)
Supplement: vbag077_Supplementary_Data [file vbag077_supplementary_data.zip › Supplementary_Note_1_Model.pdf]

Supplementary Note 1: Statistical model and implementation

We present a statistical model for assessing methylase activity in prokaryotic genomes. The input data of the model consist of the genomic sequences of one or multiple prokaryotic isolates, a set of candidate methyltransferases with known target specificities (i.e. we assume that it is known which genomic locations are methylated by each input methyltransferase, if it is present and active), and raw Nanopore sequencing data in POD5 format for each isolate. As a first step, the Nanopore sequencing data are aligned and basecalled in a methylation-aware fashion, yielding, for each 'C' or 'A' genomic position, the total number of aligned sequencing reads and the number of aligned sequencing reads carrying a 4mC-, 5mC- or 6mA-methylated base (separately recorded for the different methylation types and only for the strand that the underlying 'C' or 'A' base is observed on).

We assess methylase activity by modeling observed genome-wide methylation patterns as resulting from the activity level and target sites of the supplied candidate methyltransferases in the analyzed genome, employing a regularized logistic regression (glmnet) framework. Our model can be applied in isolate-specific mode, in which the data of each isolate are analyzed independently, or in cross-isolate mode, in which a joint statistical analysis of all isolates is carried out; even in cross-isolate mode, however, the activity of candidate methyltransferases is always assessed in an isolate-specific manner. In the following, we describe the isolate-specific mode; the cross-isolate mode follows as a generalization. Furthermore, 4mC, 5mC, and 6mA methylation are always analyzed independently.

Model definition

In our statistical analysis, the observed data are defined as the methylation status of all aligned read bases across all relevant positions in the input genome; we model the probability that an individual aligned read base is methylated as depending on the activity of the methylases targeting the corresponding genomic location, local sequence context, and the overall methylation level of the analysed isolate. Formally, let P denote the set of relevant genomic positions in the input genome (i.e., all 'C's when assessing 4mC or 5mC methylation, and all 'A's when assessing 6mA methylation); each element $p \in P$ comprises a contig identifier, a position, and strand information, specifying which strand of the input genome the relevant 'A' or 'C' base is present on. Each $p \in P$ is covered by a set B_p of aligned read bases from reads aligned to the same strand as p; $\bigcup_{p \in P} B_p$ is the set of all aligned read bases at all relevant positions in the complete genome. The methylation status of all aligned read bases in $\bigcup_{p \in P} B_p$ is a binary random variable, and, for a $b \in B_p$ at a

specific position p, we model the probability of b being methylated using glmnet (Friedman et al., 2010), employing a linear predictor of the form (regularization, with α = 1, is not explicitly shown):

$$\eta_p = \mu + \sum_{m \in M} \text{target\_site}(p,m) \times \beta_m + \sum_{c \in C} \text{context\_match}(p,c) \times \beta_c$$

, where

- μ is an intercept term capturing the overall methylation level of the analyzed isolate (independent of sequence context and methylase activity);
- M is the set of all considered methylases and $\text{target\_site}(p,m)$ is an indicator function that is 1 if and only if the position p is targeted by methylase $m \in M$, and 0 otherwise (see below for details on $\text{target\_site}(p,m)$);
- β_m is the effect of methylase m on positions recognized by the methylase;
- C is a set of genomic contexts ($C = \{'AA', .., 'TT'\}$).
- $\text{context\_match}(p,c)$ is an indicator function that is 1 if any only if the base to the left of p (in the direction of p's strand) is equal to the first base of c and the base to the right of p is equal to the second base of c, and 0 otherwise;
- β_c is the effect of the sequence context c on observed methylation levels.

The strength of regularization is determined by 10-fold cross-validation, carried out with glmnet's cv.glmnet function. To avoid collinearity, methylases with identical $\text{target\_site}(p,m)$ for all $p \in P$ are merged prior to application of glmnet.

Methylase target site recognition motifs and definition of $\text{target\_site}(p,m)$

We assume that the target site specificity of each analysed methyltransferase can be represented by a set of target site recognition motifs. Target site recognition motifs may contain IUPAC ambiguity characters and we require that each target site recognition motif contain exactly one marked base ("methylation target site"), indicating which specific position within the motif is methylated by the methyltransferase (for example, the target site recognition motif CCW^{5mC}CG would imply that the third 'C' in every genomic location that matches the pattern CCWCG is 5mC-methylated). The set of target sites for a given methylase and genome can be determined by finding all genomic locations matching any one of the target site recognition motifs of the methylase in the genome, and by marking the genomic locations corresponding to the base marked as the "methylation target site" in the corresponding genomic hit to the target site recognition motif. Multiple target site recognition motifs per methylase are required because some methylases act on more than one base within the same genomic context; this is the case e.g. for methylases that modify bases on both strands of the genomic loci identified by the target site recognition motif. For example, M.Eco1655I in the isolate *E.*

coli recognizes the motif A^{6mA}ACNNNNNNGTGC while also modifying the complementary counterpart GC^{6mA}ACNNNNNNGTT.

Formally, let M denote the set of putative methyltransferases present in an isolate; each element $m \in M$ is associated with a label $\text{label}(m)$ that specifies the name of the methyltransferase and a set $\text{methylation\_motifs}(m)$, specifying the sequence motifs of positions methylated by the methyltransferase (if it is active). Within each element $t \in \text{methylation\_motifs}(m)$, exactly one position is marked. Define a function $\text{target\_site*}(p,t)$ that is 1 if and only if the genomic position p matches the marked base in $t \in \text{methylation\_motifs}(m)$. $\text{target\_site}(p,m)$ is then defined as 1 if and only if $\bigvee_{t \in \text{methylation\_motifs}(m)}(\text{target\_site*}(p,t) = 1)$ is true.

In MPore, M is populated based on a homology search against REBASE and based on information present in REBASE. The majority of REBASE methylase entries comprise exactly one target site recognition motif with one defined methylation target site; these are represented as one $m \in M$ with a single associated $\text{methylation\_motifs}(m)$. A smaller number of REBASE methyltransferase entries are associated with more than one target site recognition motif that each have one defined methylation target site (see the M.Eco1655I example discussed above); these are represented as one $m \in M$ with multiple associated $\text{methylation\_motifs}(m)$. Finally, some REBASE methyltransferase entries specify a target site recognition motif, but no information is provided on which specific base(s) within the motif are methylated by the methylase, violating the requirement that each target site recognition motif associated with an $m \in M$ contain exactly one "methylation target" base. Methylases with $k > 1$ potential methylation target sites (according to REBASE) are therefore represented by k independent entries in M that are each associated with a unambiguously defined one-base methylation target site (for example, the recognition motif of the 4mC methyltransferase M.PspGI is CCWGG, implying that it may methylate any of the motif's two C bases; in M, this methylase is represented by two independent entries, one labelled "M.PspGI-C1" with target site recognition motif ^{4mC}CCWGG, and another one labelled "M.PspGI-C2" with target site recognition motif C^{4mC}CWGG).

Cross-isolate analysis

Cross-isolate analysis is a generalization of isolate-specific analysis. Specifically, in cross-isolate analysis mode:

- the set P of relevant genomic positions becomes the set of relevant genomic positions in all included isolate genomes; in addition to information on contig identifier, position, and strandedness, the individual elements $p \in P$ also carry information on the source genome of each p.

- the linear predictor in cross-isolate mode is extended with an isolate-specific intercept term μ_i for $i = 1 \ldots I$, where I is the total number of included isolates and isolate(p) indicates which isolate position p emanates from:

$$\eta_p = \mu + \mu_{\text{isolate(p)}} + \sum_{m \in M_{\text{isolate(p)}}} \text{target\_site}(p,m) \times \beta_m + \sum_{c \in C} \text{context\_match}(p,c) \times \beta_c$$

The set $M_{\text{isolate(p)}}$ is defined as the set of methylases present in the isolate $i = \text{isolate(p)}$; if the sets of two isolates, e.g. M_1 and M_2, contain entries with the same label, they are still treated as separate enzymes with individual coefficients (this is motivated by, first, the fact the activity level of an enzyme can differ between isolates, and, second, that M is populated by homology search against REBASE; the fact that two loci in two different isolate genomes show homology to the same REBASE entry does not imply that the two loci encode exactly the same enzyme).

By contrast, the β_c are shared between isolates; this is based on the assumption that Nanopore methylation detection biases that depend on local sequence context are likely shared between isolates.

References

Friedman, J., Hastie, T., & Tibshirani, R. (2010). Regularization Paths for Generalized Linear Models via Coordinate Descent. *Journal of Statistical Software*, *33*(1). https://doi.org/10.18637/jss.v033.i01
