# Supplementary material for: MPore: database-driven identification of active methyltransferases in prokaryotic genomes from nanopore sequencing
Source: Bioinform Adv. 2026 Mar 24;6(1):vbag077. doi: 10.1093/bioadv/vbag077 (PMC13189711; doi:10.1093/bioadv/vbag077)
Supplement: vbag077_Supplementary_Data [file vbag077_supplementary_data.zip › Supplementary_Note_2_Evaluation.pdf]

Supplementary Note 2: Evaluation

Software versions

The evaluation experiments were carried out with:

- Dorado V.0.8.0-linux
- Modkit V.0.5.0-linux
- BLASTP V.2.16.0+
- PROKKA V.1.14.6
- Python V.3.9.23
- R V.4.3.1

Bacterial monocultures dataset (Heidelbach et al., 2024)

The bacterial monocultures dataset comprises data on the presence of methyltransferases (n = 35 enzymes, 34 of which are present in the REBASE (Roberts et al., 2023) gold-standard dataset) and their associated target site recognition motifs (n = 35 motifs) for 10 isolates of different bacterial species.

Data

ID	Species	Assembly length	Average read length	Coverage	Assembly accession	Pod5 accession
1	*Cellulophaga lytica*	3,766,259	5321	17.9x	14512294	MICROBEMOD-DATA-NOV2023
2	*Desulfobacca acetoxidans*	3,282,536	7700	216.9x	14512294	ERR14125640
3	*Escherichia coli*	4,640,168	11185	437.1x	10964193	ERR12997173
4	*Kangiella aquimarina*	2,687,146	3708	744x	14512294	MICROBEMOD-DATA-NOV2023
5	*Meiothermus ruber*	3,097,467	9744	728.2x	10964193	ERR12997176
6	*Pelobacter carbinolicus*	3,665,893	9553	197.4x	14512294	ERR14125708
7	*Shewanella oneidensis*	5,135,162	3694	127.6x	14512294	MICROBEMOD-DATA-NOV2023

8	*Sphaerobacter thermophilus*	3,993,764	9704	186.4x	14512294	ERR14125702
9	*Thermanaerovibrio acidaminovorans*	1,848,474	7563	385.7x	14512294	ERR14125689
10	*Zymomonas mobilis*	2,099,363	4530	32.8x	14512294	MICROBEMOD-DATA-NOV2023

The assemblies can be downloaded from `https://zenodo.org/records/` with their specific accession number. The Pod5 data for *Cellulophaga lytica*, *Kangiella aquimarina*, *oneidensis* and *Zymomonas mobilis* can be downloaded using command:

```
aws s3 cp --recursive s3://cultivarium-sequencing/MICROBEMOD-DATA-NOV2023/pod5/
```

For the other samples, Pod5 data can be downloaded using the following links:

Species	Download links (ftp SRA)
Meiothermus ruber	ftp.sra.ebi.ac.uk/vol1/run/ERR129/ERR12997176/rawdata_meiothermus_ruber_strain_DSM1279_01.tar.gz
Desulfobacca acetoxidans	ftp.sra.ebi.ac.uk/vol1/run/ERR141/ERR14125640/rawdata_Desulfobacca_acetoxidans_DSMZ11109_01.tar.gz
Escherichia coli	ftp.sra.ebi.ac.uk/vol1/run/ERR129/ERR12997173/rawdata_ecoli_K-12_MG1655_01.tar.gz
Pelobacter carbinolicus	ftp.sra.ebi.ac.uk/vol1/run/ERR141/ERR14125708/rawdata_Pelobacter_carbinolicus_DSMZ2380_01.tar.gz
Sphaerobacter thermophilus	ftp.sra.ebi.ac.uk/vol1/run/ERR141/ERR14125702/rawdata_Sphaerobacter_thermophilus_DSMZ20745_01.tar.gz
Thermanaerovibrio acidaminovorans	ftp.sra.ebi.ac.uk/vol1/run/ERR141/ERR14125689/rawdata_Thermanaerovibrio_acidaminovorans_DSMZ6589_01.tar.gz

Enzyme- and motif-specific validation

After identifying methyltransferase candidates by homology search against REBASE, the statistical model of MPore combines enzymes with identical target specificities to avoid collinearity. A single detection of MPore therefore comprises the names of one or multiple methyltransferase enzymes as well as the associated target site recognition motifs.

The bacterial monocultures dataset comprises ground truth information at the level of enzymes (which methlytransferases are present in the isolates) and at the level of methylation motifs (what are the target site recognition motifs of the present methyltransferases). One enzyme present in the bacterial monocultures dataset (M.SonORF3004P with the target site recognition motif G^{6mA}ATC, present in the *Shewanella oneidensis* genome) is not present in the REBASE gold-standard dataset and can therefore not be detected by MPore; REBASE, however, comprises multiple entries with the same target size recognition motif G^{6mA}ATC. We therefore separately evaluate the performance of MPore at the enzyme and at the motif level (note that, in the results presented in the paper, *Shewanella oneidensis* is part of the reference dataset that was used for setting the activity threshold; the isolate does therefore not appear in the "validation" part comprising 7 isolates, which are used for benchmarking sensitivity and precision; see Section "Determination of enzyme activity threshold" below for details).

For the enzyme-level evaluation,

- a single enzyme present in the ground truth dataset is counted as found by MPore if its name appears among the names of the enzymes found and classified as active by MPore.
- for calculating precision, a single detection by MPore is counted as present in the ground truth if any of the enzyme names associated with the detection are present in the ground truth dataset.

For the motif-level evaluation,

- for calculating recall, a single motif in the ground truth dataset is counted as found by MPore if there is an MPore detection with the same motif and it is classified as active.
- for calculating precision, a single detection by MPore is counted as present in the ground truth if the associated motif is present in the ground truth dataset.

Determination of enzyme activity threshold

The statistical model of MPore determines an estimated activity level (i.e., a β coefficient) for each identified methyltransferase candidate. To determine a threshold on the estimated β coefficient that distinguishes between true- and false-positive detections, we used a subset of 3 bacterial monocultures (*E. coli*, *S. oneidensis*, *T. acidaminovorans*). Optimal separation was achieved at $\beta^* = 2.021$ (i.e. a detection by MPore is only counted as "active" if its associated β coefficient is > 2.021), with 100 % enzyme-level recall and 98 % enzyme-level precision in the 3 isolates (see Figure 1 below).

Reported evaluation metrics on the bacterial monocultures in the main paper exclude the 3 isolates used for determining the β threshold.

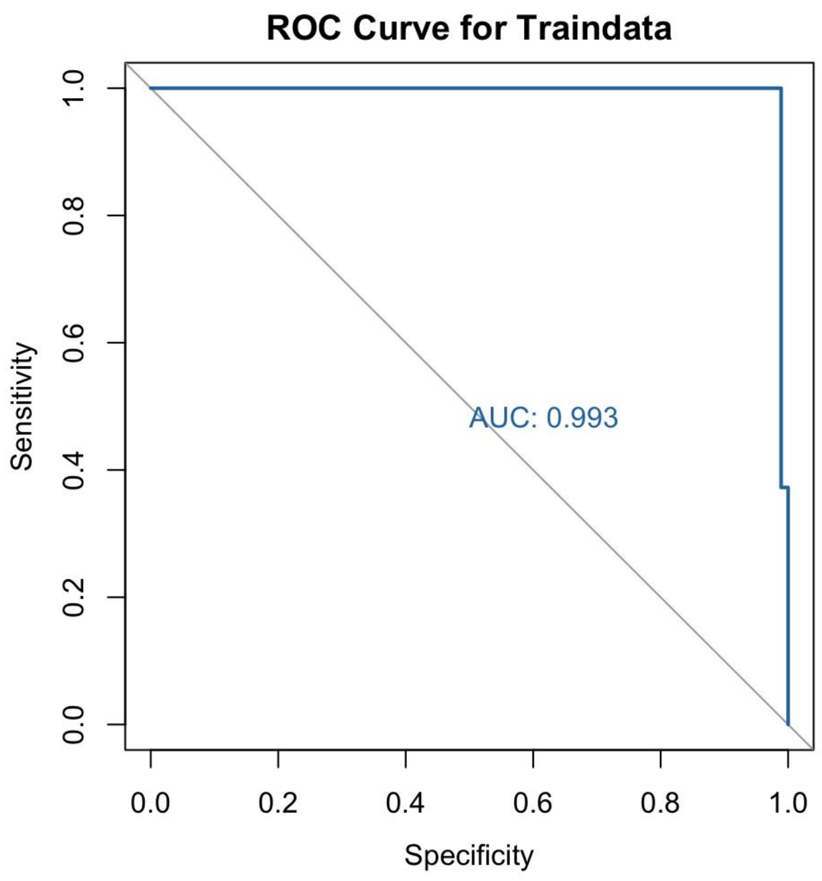

Figure 1. ROC across three monocultures. The figure shows the Receiver Operating Characteristic curve for the training data set consisting of the three monocultures (*E. coli*, *S. oneidensis*, *T. acidaminovorans*). Sensitivity and specificity were measured at the enzyme level.

Comparison of isolate-specific and cross-isolate analysis modes

On the bacterial monocultures dataset, isolate-specific and cross-isolate analysis modes produced very similar results with respect to the number of overall detections and their associated β coefficients (Figure 2 below).

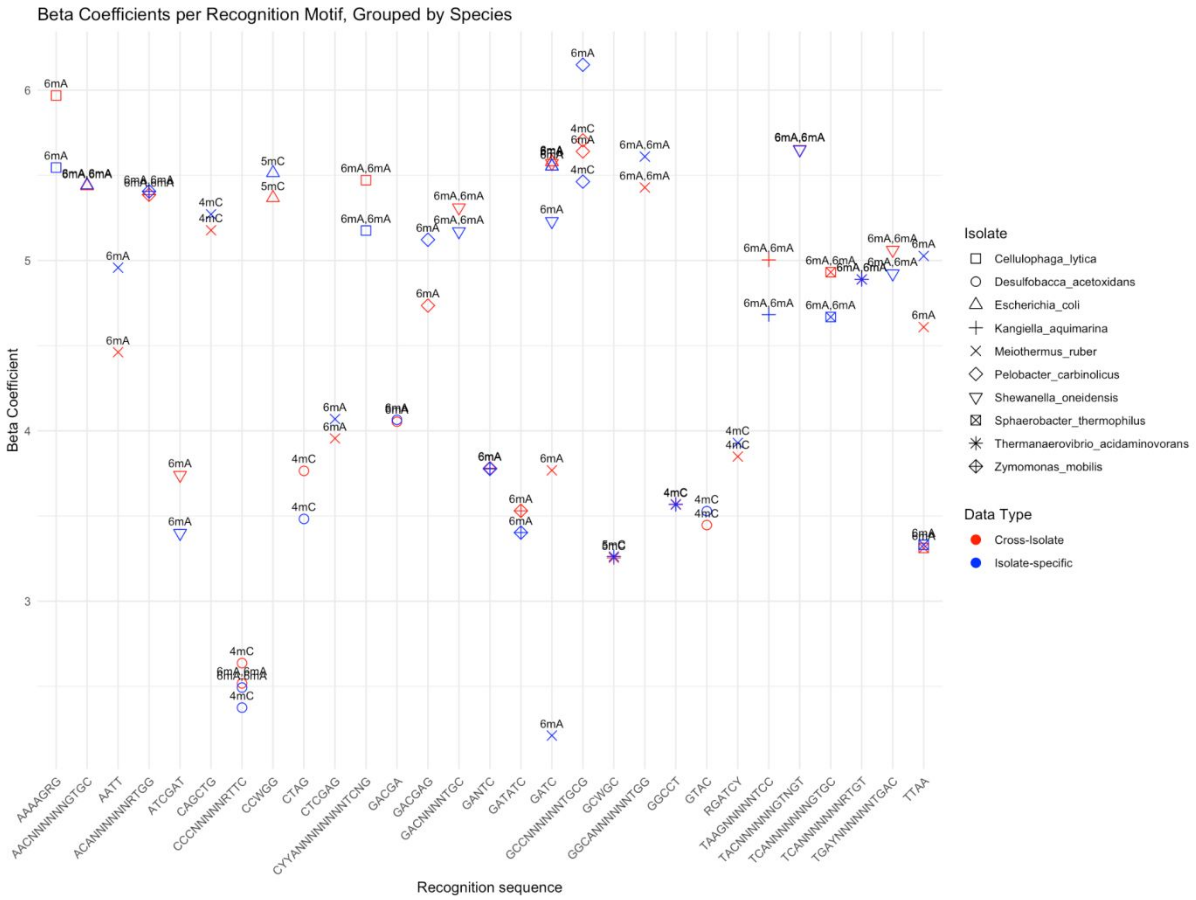

Figure 2. Estimated single-isolate and cross-isolate beta coefficients for the bacterial monocultures dataset. The figure shows estimated beta coefficients for the MPore detections (i.e., after combining enzymes with identical target specificities) across the benchmark dataset. Cross-isolate estimates are shown in red, isolate-specific estimates in blue. The isolates are visually distinguished with different shapes.

Mycoplasma hominis dataset (Vogelgsang. et al. 2023)

The bacterial monocultures dataset comprises 6 *Mycoplasma hominis* isolates, which were part of a previously published in-depth investigation of methylation patterns in *M. hominis* (Vogelgesang et al. 2023). To evaluate MPore on this dataset, we compared the detections of MPore to the computational and wet-lab-based validation results described in Vogelgesang et al. 2023. For the analysis of the *M. hominis* data, we applied the same $\beta^* = 2.021$ that was also used for the analysis of the bacterial monocultures dataset (i.e. a detection by MPore was only counted as “active” if its associated β coefficient was > 2.021).

Data

ID	Isolate name	Assembly length	Average length	Coverage	Accession assembly	Accession pod5
1	12256U	835,540	4059	663.2x	https://osf.io/czrbt/	PRJNA429440
2	8958VA	680,851	7556	3265.5x	PRJNA429440	PRJNA429440
3	A136	696,338	767	4230.2x	PRJNA429440	PRJNA429440
4	SP10291	750,518	1819	267.8x	PRJNA429440	PRJNA429440
5	Sp2565	712,781	4208	3952.6x	PRJNA429440	PRJNA429440
6	VO31120	681,374	5806	5599.3x	PRJNA429440	PRJNA429440

Assemblies and raw sequencing data for *M. hominis* can be downloaded from BioProject PRJNA429440.

Comparison of isolate-specific and cross-isolate analysis modes

On the *M. hominis* dataset, isolate-specific and cross-isolate analysis modes produced broadly similar results with respect to the overall number of detections and their associated β coefficients (Figure 3 below).

Of note, however, the cross-isolate and isolate-specific execution modes of MPore different with respect to detections (instances in which the estimated beta coefficient was above the threshold value of 2.021) in three isolates:

- Isolate SP10291: M.TacI with motif (motif:TC^{6mA}ANNNNNNNRTGT/AG^{6mA}AYNNNNNNNTGA) was classified as active in isolate-specific mode, whereas its estimated activity remained below the threshold of 2.021 in cross-isolate mode.

  Inspection of the empirical methylation at target sites of M.TacI showed high methylation at individual positions, but low methylation levels at the majority of target positions (Figure 4); the empirically observed methylation levels were therefore more consistent with the cross-isolate results of MPore.

- Isolates A136 and VO31120: M.HpyD27I with motif: ^{5mC}CCTC/G^{6mA}AGG was found to be inactive with respect to 5mC methylation in single-isolate mode, whereas it was found to be active in cross-isolate mode for the two isolates.

  Inspection of the empirical methylation at target sites of M.HpyD27 in the two isolates overall high methylation levels, consistent with MPore cross-isolate results.

The exact estimates for cross-isolate vs. isolate specific mode for these three isolates are listed in Supplementary Table 5.

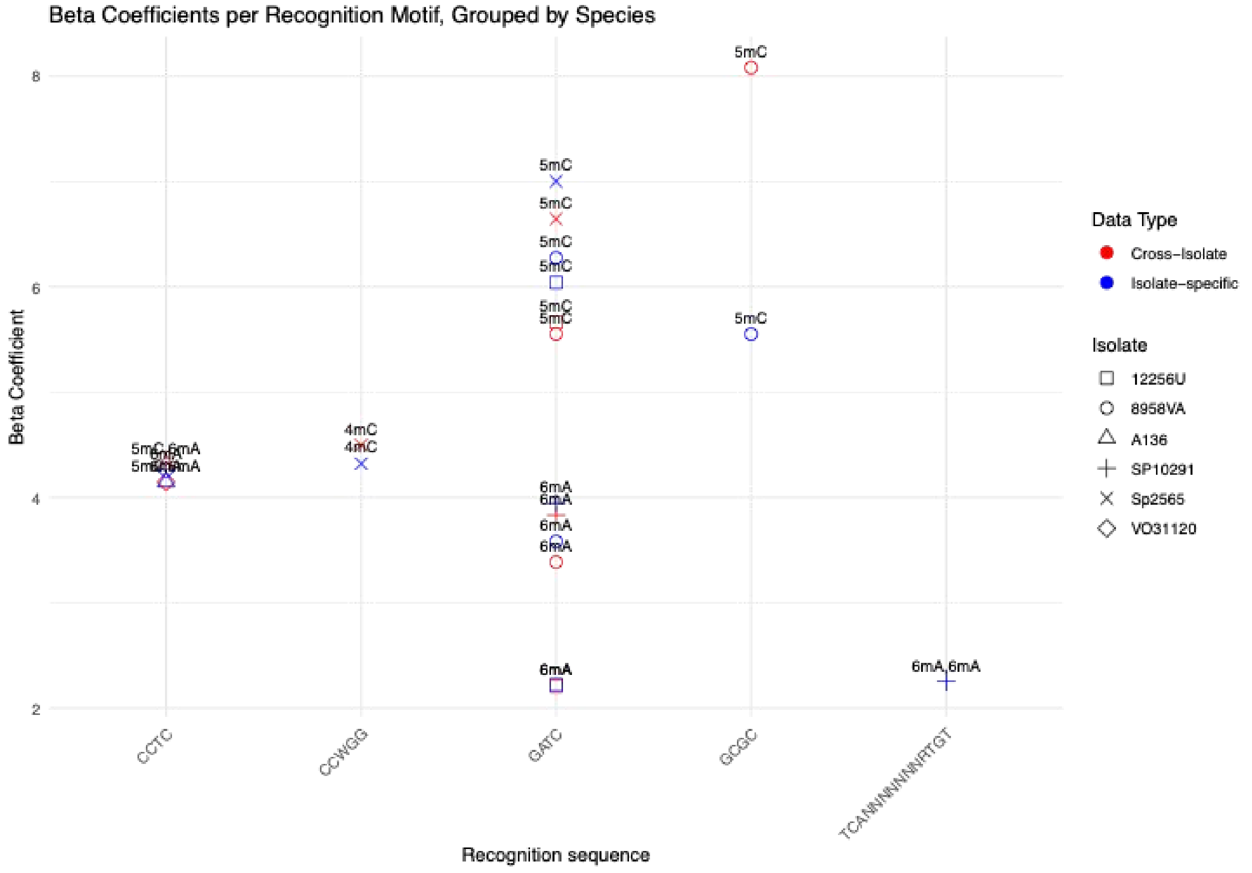

Figure 3. Estimated single-isolate and cross-isolate beta coefficients for *M. hominis* dataset. The figure shows estimated beta coefficients for the MPore detections (i.e., after combining enzymes with identical target specificities) across the benchmark dataset. Cross-isolate estimates are shown in red, isolate-specific estimates in blue. The isolates are visually distinguished with different shapes.

M.TacI (AC^{6mA}AYNNNNNNNTGA)

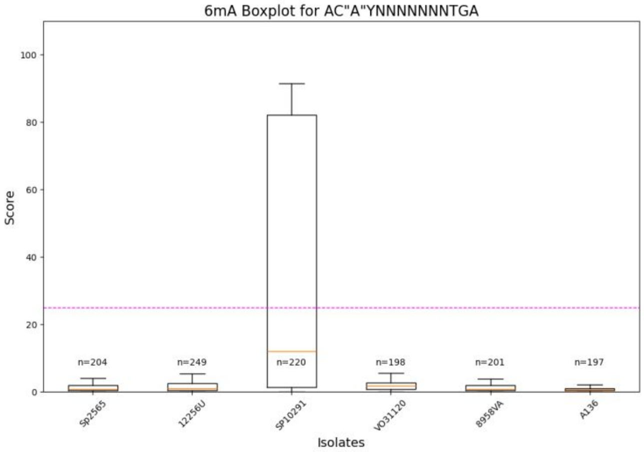

Enzyme	Uniquemethylation	Motif	MethylationType	Strand	Position	Sp2565	12256U	SP10291	VO31120	8958VA	A136
M.TacI	M.TacI	TCANNNNNNNRTGT	6mA	-	3	2.12e-65 (204), ß=-0.59	6.03e-65 (249), ß=-0.24	2.77e-65 (220), ß=1.86	2.77e-65 (198), ß=0.10	1.09e-128 (201), ß=-0.13	2.12e-65 (197), ß=-1.13

Figure 4. Nanopore methylation signal for AC^{6mA}AYNNNNNNNTGA. The figure shows the nanopore methylation signal for the motif AC^{6mA}AYNNNNNNNTGA across 6 *M. hominis* isolates. Below the boxplot, entries show MPore detections which explain the observed signal.

M.HpyD27I (^{5mC}CCTC)

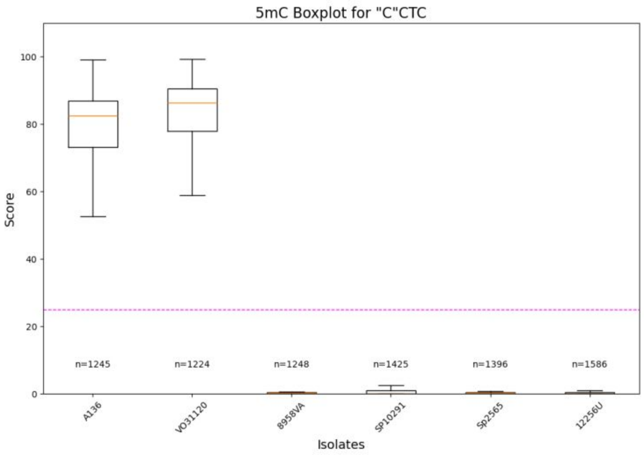

Enzyme	Uniquemethylation	Motif	MethylationType	Strand	Position	A136	VO31120	8958VA	SP10291	Sp2565	12256U
M.HpyD27I	M.HpyD27I	CCTC	5mC	+	1	0.00e+00 (1245), ß=1.98	0.00e+00 (1224), ß=1.92	nan	nan	nan	nan
M.CpeAIII	M.CpeAIII	GATC	5mC	+	4	nan	nan	7.33e-45 (20), ß=7.21	nan	1.70e-45 (20), ß=8.17	9.96e-38 (26), ß=7.20

Figure 5. Nanopore methylation signal for ^{5mC}CCTC. The figure shows the nanopore methylation signal for the motif ^{5mC}CCTC across 6 *M. hominis* isolates. Below the boxplot, entries show MPore detections which explain the observed signal

H. pylori benchmark dataset

Subsequently, we benchmarked MPore against motif-based approaches, including Modkit (https://github.com/nanoporetech/modkit), Snappy (Konanov et al., 2025) and Nanomotif (Heidelbach et al., 2024) , using two Helicobacter pylori datasets characterized by a diverse and complex methylome from the ONT methylation benchmark dataset.

The ONT benchmark dataset comprises two *H. pylori* samples J99 and 26695 together with their respective reference genomes, all of which are publicly available. Raw Pod5 signal data can be downloaded via the following commands:

```
aws s3 cp --no-sign-request s3://ont-basemod-benchmark-data/Raw/pod5/bacteria/HP26695_WT_5kHz/
aws s3 cp --no-sign-request s3://ont-basemod-benchmark-data/Raw/pod5/bacteria/ HPJ99_WT_5kHz/
```

Corresponding reference genomes are available at:

```
aws s3 cp --no-sign-request s3://ont-basemod-benchmark-data/Analysis/Reference/ hpylori_26695.fa.gz
aws s3 cp --no-sign-request s3://ont-basemod-benchmark-data/Analysis/Reference/ hpylori_J99_ATCC700824.fa.gz
```

ID	Isolate name	Assembly length	Average length	Coverage
1	HP26695	1.667.867	3.657	103x
2	HPJ99	1.643.831	14.435	107x

This setup allows for a comprehensive evaluation of MPore relative to de novo based tools in a controlled benchmark context.

As a ground truth for both datasets, we relied on an independent study (Krebes et al., 2014) which analyzed the diverse and complex methylomes of H. pylori strains J99 and SP26695 at single-base resolution using Single Molecule Real-Time (SMRT) sequencing. Across these strains, they identified 39 methylated sequence motifs and characterized the corresponding MTases through functional inactivation, frameshift corrections, and cloning and expression experiments. For our analysis, we applied the same β-threshold of 2.021 for active MTases in MPore and compared the results to the de novo approaches reported in Supplementary Table 6.

Across both strains, MPore employing a database-driven approach and statistical assessment of activity achieved the highest performance metrics with PPV 0.95 and recall 0.93 (for detailed analysis see table 1 in manuscript). All tools exhibited false-positive detections some motifs, such as “GWCAY,” were consistently identified across approaches, highlighting the value of combining bioinformatic predictions with experimental validation. MPore achieved the highest positive predictive value (PPV) and recall in the context of the complex and diverse *H. pylori* methylomes. Nevertheless, certain enzymes identified by Krebes et al. (2014) were not detected by MPore, reflecting limitations in identifying MTases and motifs absent from the REBASE database. Despite these limitations, the present analysis demonstrates that MPore maintains strong performance even in complex methylome contexts, outperforming de novo approaches.

	MPore gene hits	MPore motif hits	Nanomotif motif hits	Modkit motif hits	Snappy motif hits
Total	27	34	25	25	32

HP26695	13	16	14	15	13
HPJ99	14	18	11	10	19
FP	4	3	6	22	9
	Mpore (Gene)	MPore (Motif)	Nanomotif	Modkit	Snappy
PPV	0.87	0.91	0.81	0.53	0.78
TPR (Recall)	0.69	0.87	0.64	0.64	0.82
Accuracy	0.63	0.81	0.55	0.41	0.66

Parameter setting for de novo approaches

Modified basecalling for the *Helicobacter pylori* datasets was performed using Dorado v0.8.0 in a Linux command shell. The resulting reads were aligned to the corresponding reference genomes, and the binary alignment map (BAM) files were sorted and indexed using Samtools v1.21.

Parameter settings for *de novo* approaches is the same across the different datasets (bacterial monocultures and *H.pylori* datasets).

Information regarding the methylation status at each cytosine and adenine was extracted using Modkit v0.4.1, which generated per-base information BED files containing methylation probabilities for each isolate. These BED files were then used for motif discovery motif search with Modkit under default settings. Modkit identifies modified k-mers by comparing their observed frequencies to the expected frequencies in an unmethylated background, thereby summarizing short genomic sequences (motifs) that are enriched for methylation. For detailed information, see [https://nanoporetech.github.io/modkit/intro_find_motifs.html].

Model used for dorado basecalling are set with `–modified-base-models` dna_r10.4.1_e8.2_400bps_hac@v5.0.0_4mC_5mC@v1

dna_r10.4.1_e8.2_400bps_hac@v5.0.0
dna_r10.4.1_e8.2_400bps_hac@v5.0.0_6mA@v1

Command line for dorado base and modification calling:

```
{input.dorado_path}/bin/dorado basecaller --modified-bases-models
{input.dorado_path}/lib/dna_r10.4.1_e8.2_400bps_hac@v5.0.0_4mC_5mC@v1,{input.dorado_p
ath}/lib/da_r10.4.1_e8.2_400bps_hac@v5.0.0_6mA@v1
{input.dorado_path}/lib/dna_r10.4.1_e8.2_400bps_hac@v5.0.0  {params.pod5_path} --
reference {params.reference_path} > {output.bam}
```

Command line for sorting and indexing bam files generated via dorado basecaller {output.bam}:

```
samtools sort -o {output.sorted_bam} {output.bam}
```

```
samtools index {output.sorted_bam}
```

The paths in {}-brackets are replaced with directories leading to the exact files e.g. pod5 files or installed dorado directories and models. These sorted and indexed bam files are then decoded using Modkit V.0.4.1 with its pileup function generating a browser-extensible-data (bed) file, which is a tab-separated per-base overview with features like number of reads with methylated base and total coverage per base.

Command line for Modkit pileup:

```
modkit pileup {params.bam_path} {output[0]} --ref {params.reference_path}
```

The parameter {output[0]} is a snakemake defined parameter and creating a bed file with isolate specific names for each isolate e.g. Shewanella_oneidensis.bed. These bedmethyl files are used as input for each *de novo* based approach and MPore.

Modkit motif analysis

Motif discovery was performed with the `motif search` function of Oxford Nanopore technologies tool Modkit (version 0.4.1).

The following command was applied to each bacterial isolate using isolate-specific reference assemblies and BED methylation files (see section modkit pileup):

```
Bedmethyl=/path/to/bedmethyl.bed

Ref=/path/to/reference.fasta

Modkit motif search -i ${Bedmethyl} -r ${Ref} -o /path/to/output/motifs.tsv --log /path/to/Output/Modkit_find_motifs_log.txt
```

Of note, this command was used as loop across the set of all bacterial isolates with isolate-specific reference.fasta files, output directories and Bedmethyl files. For the isolate *Sphaerobacter thermophilus* the parameter –search-timeout was set to 15m.

Command line for *Sphaerobacter thermophilus*:

```
Bedmethyl=/path/to/Sphaerobacter_thermophilus.bed

Ref=/path/to/Sphaerobacter_thermophilus.fasta

Modkit motif search -i ${Bedmethyl} -r ${Ref} --search-timeout 15m -o /path/to/output/motifs.tsv --log /path/to/Output/Modkit_find_motifs_log.txt
```

This parameter constrains the time spent on the seed extension, convergence, and motif ambiguity resolution to 15 minutes.

All parameters were not changed from their default setting besides listed for *Sphaerobacter thermophilus*.

Nanomotif analysis

Motif discovery using Nanomotif (version 1.0.2) was performed according to the developer documentation.

Input files consisted of:

- Modkit-generated methylation pileup BED files
- Corresponding reference genome assemblies
- Contig-to-bin mapping files

Contig bin files were generated such that each isolate and its contigs were assigned to a single bin. Reference contig names were restricted to IUPAC-compliant characters as required by Nanomotif.

Nanomotif constructs position-probability-matrices (PPMs) for modified and unmodified contexts using per-base methylation calls and identifies enriched sequence patterns based on PPM score differences.

Motif discovery was performed using:

```
nanomotif motif_discovery $ASSEMBLY $PILEUP -d $BINS --out $OUT
```

All parameters were left at default settings, and no adjustments were applied.

Snappy analysis

Motif discovery using Snappy (version 0.1.6) was applied to the same datasets.

Snappy identifies methylation motifs by combining basecaller modification probabilities with graph-based enrichment algorithm to detect motifs enriched in methylated target sequences compared to the unmethylated genomic background.

Input data consisted of Modkit methylation BED files and corresponding reference genome assemblies.

Motif discovery was performed using:

```
snappy -mk_bed $PILEUP -genome $ASSEMBLY -outdir $OUT
```

Genome assembly generation from aligned reads was not required because reference assemblies were already available.

All analyses were performed using default parameters.

For additional implementation details, see the official Snappy repository: https://github.com/DNKonanov/ont-snappy
